# Commentary: The quantitative paradigm and the nature of the human mind. The replication crisis as an epistemological crisis of quantitative psychology in view of the ontic nature of the psyche

**DOI:** 10.3389/fpsyg.2025.1649683

**Published:** 2025-08-13

**Authors:** Mario S. Staller, Swen Koerner

**Affiliations:** ^1^Department of Police, University of Applied Sciences of Police and Public Administration North Rhine-Westphalia, Cologne, Germany; ^2^Department of Training Pedagogy and Martial Research, German Sports University Cologne, Cologne, Germany

**Keywords:** systems theory, Niklas Luhmann, reflexivity, theory crisis, replication crisis, ontology, self-referential systems, psychic systems

In their article, (Mayrhofer et al. 2024) make a compelling claim: the replication crisis in psychology is not merely a methodological or institutional challenge, but reflects a deeper epistemological rupture. Drawing on classical philosophy of science, they argue that the problem may lie in a fundamental mismatch between the epistemic structures of quantitative psychology and the ontic nature of the human psyche. This perspective opens a discussion beyond statistical reform and procedural refinements. It invites us to reconsider the very theoretical architecture upon which psychology as a science is built.

From our perspective, a key strength of the article lies in the analysis of the “ontic-epistemic mismatch”, that produces inconsistent and irreproducible findings, because of a structural incoherence between what psychology studies and how it studies it. However, this theoretical tension becomes observable in the architecture of the authors' argument itself. While the authors problematize that psychology may misrepresent the nature of its object, they do so from within a theory architecture that presupposes a stable distinction between observer and observed. In their analysis, psychology appears as a science struggling to grasp a resistant object. The risk of solutions derived from within this ontological framework is that the proposed solution addresses the content of the problem while preserving the very form that produces it. As long as the psyche is conceptualized as an entity with hidden internal properties, the epistemic logic remains one of access, adequacy, and representation.

We propose an alternative approach: one that shifts focus from the nature of the object to the structure of observation itself. Luhmann's systems theory (Luhmann, 1996, 2012, 2013) provides a pathway for such a structural reframing. While his contribution has been extensively acknowledged in the field of sociology, his “supertheory” (Moeller, 2011) has the potential to be applied in other fields as well. While some contributions have been made (e.g., Fuchs, 2003, 2021; Luhmann, 1985; Simon, 1993, 2004)—a comprehensive theory of psychic systems built explicitly on this architecture remains underdeveloped.

Instead of redefining what the psyche is (quantitative or not), a system-theoretical approach reconfigures the form of observation itself. It no longer seeks to access a stable object but treats the psyche as a system constituted by its own operations. From this perspective, the core question shifts from what the psyche is to how it produces itself through meaning-based, autopoietic processes (Luhmann, 1985). If we take the system as the unit of analysis, the replication crisis reflects the blind spots of the observing system. Psychological research, when shaped by a subject-object epistemology, assumes that mental phenomena can be stabilized through controlled observation. In contrast, a system-theoretical perspective maintains that experience is not directly observable. The psyche generates its own activity through contingent structural couplings, not externally accessible states. From this vantage point, the failure to replicate does not signal methodological failure, but points to a misalignment in theory architecture. The expectation that psychological systems will produce invariant data overlooks their operational closure, context-dependence, and self-referential dynamics. In doing so, the scientific system observes another operatively closed system—the psyche—while neglecting to observe the form and contingency of its own operations.

In this light, many of the arguments put forward by (Mayrhofer et al. 2024) can be reread: their insights into the ontic-epistemic mismatch gain additional meaning when understood not as a failure to describe the object (the psyche) correctly, but as an effect of the observational form. Non-replicability may here as well serve as an indicator of theoretical misalignment—what others have referred to as a “theory crisis” in psychology (Eronen and Bringmann, 2021; Oberauer and Lewandowsky, 2019). However, we wish to emphasize that the shift we propose concerns the form of observation itself, not merely the content produced within the prevailing observational logic of mainstream psychology. This observational transition—from a subject-object based epistemology to a systems-theoretical one—has far-reaching consequences. It would reposition psychology from a science about the psyche to a science that observes how psychic systems process experience. The goal would shift from explanation to the structural reconstruction of psychic operations. We are fully aware that such a proposal may be difficult to assimilate. However, the radicality (Moeller, 2011) with which Luhmann initiated a comparable shift in sociology continues to produce far-reaching theoretical and empirical insights to this day.

Our proposal introduces at least three major shifts within the logic of psychological theorizing. These shifts build upon one another by progressively reframing how psychology observes:

- A shift from representation to contingent observation: Observation is no longer treated as a neutral window onto external reality but as a system-bound operation shaped by its own distinctions. Every observation includes what it excludes. Rather than aiming for accurate representations, psychology in this view becomes reflexively aware of how its own distinctions shape what becomes empirically visible.
- A shift from subject to system: Based on this reframed observational form, the conceptual subject becomes incompatible with the logic of operatively closed systems. Paradoxically, it is only by letting go of the subject as a theoretical foundation that psychology can approach the psyche as a self-generating system of experience. What was once attributed to a unified subject becomes re-described as systemic recursion: the psyche experiences itself through the difference it produces.
- A shift from empirical primacy to structural interdependence: Once observation is understood as form-bound and the psyche as an operatively closed system, the relation between theory and empiricism must also be reconceived. Theory and empiricism are not hierarchically ordered but structurally interdependent. Theory defines the distinctions that render empirical observation possible, while empirical findings reveal the blind spots and contingencies of those distinctions. Empiricism in this view does not confirm or refute theory—it stimulates it.

From a systems-theoretical view, this moment invites not just better answers, but different questions—specifically about the form of observation itself. Our proposal is not a resolution, but a reframing—one that remains contingent, yet opens space for alternative descriptions of psychology and the systems it brings into form through its own operations.
